# Charge balance calculations for mixed salt systems applied to a large dataset from the built environment

**DOI:** 10.1038/s41597-022-01445-9

**Published:** 2022-06-17

**Authors:** Sebastiaan Godts, Michael Steiger, Scott Allan Orr, Tim De Kock, Julie Desarnaud, Hilde De Clercq, Veerle Cnudde

**Affiliations:** 1 Monuments Lab, Royal Institute for Cultural Heritage (KIK-IRPA), Brussels, Belgium; 2 Antwerp Cultural Heritage Sciences, ARCHES, University of Antwerp, Antwerp, Belgium; 3 Department of Geology, PProGRess, Ghent University, Ghent, Belgium; 4 Department of Chemistry, University of Hamburg, Hamburg, Germany; 5 Institute for Sustainable Heritage, University College London (UCL), London, United Kingdom; 6 Renovation & Heritage Lab, Belgium Building Research Institute (BBRI), Saint-Gilles, Belgium; 7 Department of Earth Sciences, Utrecht University, Utrecht, The Netherlands

**Keywords:** Natural hazards, Inorganic chemistry, Phase transitions and critical phenomena, Thermodynamics

## Abstract

Understanding salt mixtures in the built environment is crucial to evaluate damage phenomena. This contribution presents charge balance calculations applied to a dataset of 11412 samples taken from 338 sites, building materials showing signs of salt deterioration. Each sample includes ion concentrations of Na^+^, K^+^, Mg^2+^, Ca^2+^, Cl^−^, NO_3_^−^, and SO_4_^2−^ adjusted to reach charge balance for data evaluation. The calculation procedure follows two distinct pathways: i) an equal adjustment of all ions, ii) adjustments to the cations in sequence related to the solubility of the theoretical solids. The procedure applied to the dataset illustrates the quantification of salt mixture compositions and highlights the extent of adjustments applied in relation to the sample mass to aid interpretation. The data analysis allows the identification of theoretical carbonates that could influence the mixture behavior. Applying the charge balance calculations to the dataset validated common ions found in the built environment and the identification of three typical mixture compositions. Additionally, the data can be used as direct input for thermodynamic modeling.

## Abstract (summary)

**Table:** 

Measurement(s)	Ion • inorganic salt
Technology Type(s)	ion chromatography
Factor Type(s)	sample location country code (SLCC) • sample city (SC) • sample (site) name • sample material (SM) • sample height (H) • Sample depth from surface of specific material (D)
Sample Characteristic - Organism	Element, Ion, or Isotope
Sample Characteristic - Environment	building
Sample Characteristic - Location	Belgium

## Background & Summary

The continuous interaction of mixed salt systems within the environment is a powerful cause of geomorphological changes, such as the deterioration of stone^1–8^, but is also important in a wide range of scientific fields^9–21^, for example, industrial processes and planetary research. With the method and data presented we focus on ion mixtures that are found in the built environment, which directly link to other fields due to their inherent origin from earth materials, polluted ground and rainwater infiltrations, interactions with pollutants in the atmosphere and general industrial chemistry^22–25^. The importance of this subject is further underlined by the complexity of salts in the decay process of porous building materials as this is directly linked to economic, industrial, and environmental issues. It also causes the intricate loss of value to cultural heritage with socioeconomic consequences^26–29^.

Therefore, salt mixtures in Belgian monuments, archeological sites and sculptures have been analyzed by the Royal Institute for Cultural Heritage (KIK-IRPA) since the early 1960’s. In 2004, KIK-IRPA started analyzing the most relevant ions found in building materials^30^: Na^+^, K^+^, Mg^2+^, Ca^2+^, Cl^−^, NO_3_^−^, and SO_4_^2−^. This led to the establishment of a unique dataset of 11412 samples from primarily deteriorating building materials. The rationale for this extensive sampling is because limited salt analysis often guided conservation scientists toward an incorrect interpretation of the problems. The most prominent issue being that the analysis of efflorescence at the surface or analysis of anions alone does not represent the true salt mixture contamination of the material. Consequently, inappropriate conservation measures were taken, leading to significant increase in material loss and costs over time.

However, the understanding of salt mixture behavior is not a straightforward task as the formation and dissolution of possible solids under changing environmental conditions is highly sensitive to very small changes in mixture composition. To aid the understanding of which solids can form several theoretical models are available that output, amongst others, specific equilibrium, mutual deliquescence, and crystallizations relative humidity events of salts in a mixture. ECOS/Runsalt^30,31^ is the most frequently used model for this purpose and requires input of the contents of the seven ions mentioned before. When modeling salt behavior, achieving charge balance between anions and cations is important. However, from ion analysis some deviation from electric neutrality is normal and is often the result of an analytical uncertainty associated with the accuracy of the equipment, calibration, or method. More importantly the imbalance can be associated with a measurement error or ions not analyzed. This paper presents a calculation procedure applied to the dataset to achieve charge balance amongst measured ion concentrations following two distinct pathways: i) considering an analytical uncertainty or, ii) the identification, quantification and removal of cations related to undetected anions. The corrections are critical to evaluate charge balance of individual samples and before data is used as input for modeling. When evaluating ions not analyzed the procedure focusses primarily on anions that are commonly found in the built environment, such as, hydroxide, bicarbonate and carbonate, and abstains from considering the rare contribution to the total charge balance by, fluoride, phosphate, oxalate, ammonia, acetate, or formate as described by Steiger & Heritage^32^, and Arnold & Zehnder^33^. However, the ions not considered in our dataset can be added and incorporated in the calculations if deemed necessary. The method addresses the need to produce improved models from measured ion concentrations that enable equilibrium behavior to be interpreted by a wide range of users. In turn, the results can aid researchers, conservators, and site managers more generally towards a better understanding of salts. The analysis of this unique dataset has the potential to significantly improve the overall understanding of salt behavior and advance conservation recommendations underpinned by scientific evidence.

## Methods

### Materials

The charge balance procedure presented is applied to ion concentrations of soluble salts found in the built environment, including the following (most common) ions: cations Na^+^, K^+^, Mg^2+^, Ca^2+^, and anions Cl^−^, NO_3_^−^, SO_4_^2−^. The current dataset includes 11412 samples analyzed with ion chromatography, thus 79884 ion values, from 338 different monuments, archaeological sites, or sculptures, further described as sites. 330 of these sites are in Belgium, while the remaining sites in the Czech Republic (4 sites), 2 in the Netherlands, 1 in Germany, and 1 in Italy. The different sites in Belgium are spread out over 186 cities. The dataset includes general information, such as, the sampling date, location, object name, material, height, and depth. More specifically, a range of different materials are included, such as, traditional lime-based mortar, cement, plaster (including wall-paintings), brick, natural stone (mainly limestone), and efflorescence. Samples were drilled (Ø 6 mm) without the use of water at different depths and heights, respectively in a wide range of intervals from the surface to 0.1, 1, 2, 3, 5 or 10 cm to a depth of maximum 20 cm in the masonry, and from the ground level to heights up to 20 meters (such as church vaults). To illustrate the spread of samples, 788 samples were taken in 25 different sites in Antwerp, in one site ‘*Museum Vleeshuis*’ 108 samples were taken in 6 different areas, at 3 different heights per area, and at each height the drill samples were taken in the limestone and lime mortar at three different depths from the surface (0–1, 1–3 and 3–5; all in cm).

The ion analysis was carried out on the filtered extract of the dry sample mass by the addition of ultra-pure water and mixed for several hours (no constant time was upheld or noted, however extensive tests in our lab showed that all salts of interest were dissolved after approximately two hours. Logically, the final content of less soluble salts is dependent on the time and the water to sample ratio. The mean sample mass of all 11412 samples was 1.026 g (median: 0.865 g) while the water content added to the samples was on average 0.1 L per g dry sample mass.

### Preparation

The raw data derived from the ion analysis is given as a concentration of each ion as milligram per liter (mg/L). To facilitate the interpretation of the ion content in the material it is common practice to present the raw ion data as a weight percentage relative to the dry sample mass. Here, this is presented as weight fraction:

1 $${w}_{i}=\frac{{c}_{i}{V}_{w}}{{m}_{s}}$$

where *w*_*i*_ is weight fraction of each individual ion, *c*_*i*_ is the concentration of the ion (mg/L), *V*_*w*_ the volume of water (L) used for the extraction of ions from the dry sample mass represented with *m*_*s*_ (mg).

This approximation assumes a liter of solution is equal to a kilogram of pure water (1.000 g/cm³). Although a correction could be applied if the solution density of each individual sample solution was known, the calculated deviation is considered negligible, as the solutions are highly diluted, thus resulting in an acceptable deviation of several tenths to hundredths of mg/L.

### Charge balance calculations

Salt mixtures are always electrically neutral, thus, any deviation from electrical neutrality must be the result of either a measurement error or ions not analyzed. Some deviation from the analysis is common, therefore, to correctly evaluate the salt content in each individual sample and facilitate the calculations of the ionic balance, the concentration of each ion is converted to equivalents per kilogram (Eq/kg). The calculation eliminates the charge difference between ions and allows direct comparison between cation and anion balance:

2 $${e}_{i}=\frac{{w}_{i}\left|{z}_{i}\right|}{M}$$

here *e*_*i*_ is Equivalents per kilogram (Eq/kg) per ion, *z*_*i*_ the absolute charge of the ion and *M* the molar mass of the ion in question (kg/mol).

With the values in equivalents per kilogram an initial analysis is carried out to determine the charge imbalance (Δ*e*), thus the charge excess between the total sum of amount of all cations (*e*_cat_) and all anions (*e*_ani_), by:

3 $$\Delta e={e}_{{\rm{cat}}}-{e}_{{\rm{ani}}}$$

**Fig. 1:**
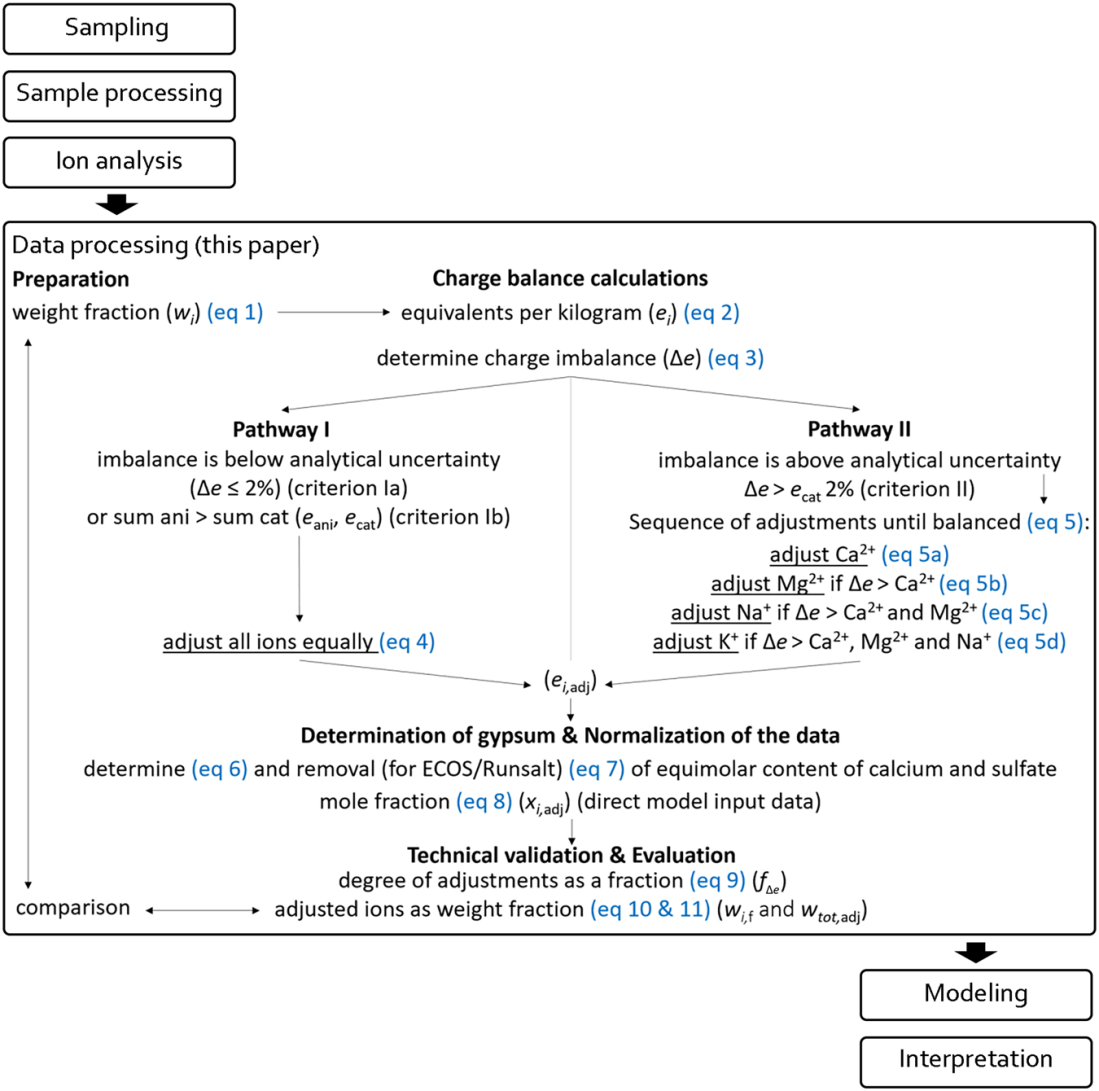
Flowchart illustrating the entire data processing sequence starting with the preparation of the data, charge balance calculations, and addressed later in the manuscript the gypsum determination, normalization of the data for modeling purposes, technical validation and evaluation section, ending with a comparison of the data before and after adjustments.

The charge balance adjustments are applied in the next equations and are based on the charge balance with a charge excess limit of 2%. This limit is dependent on the analytical method/equipment, calibration, and is ideally adjusted accordingly, further specified by Steiger and Heritage^32^. Naturally, some variation is possible, which shouldn’t reflect in the final evaluation of the ion content when considering weight fraction. The data follows one of two pathways: when the charge excess limit is equal or smaller than 2% and the anion content is greater than the cation content pathway I is followed, if it exceeds 2% pathway I is skipped and then, the data follows pathway II, as illustrated in the flowchart (Fig. 1).

#### Pathway I

Pathway I is followed when either one of two criteria are met:

- criterion Ia: an analytical uncertainty is probable when the excess (Δ*e*) is less or equal to 2% compared to the greatest value between the total sum of amount of all anions (*e*_ani_) and all cations (*e*_cat_): $\Delta e\le \left(0.5\left({e}_{{\rm{ani}}}+{e}_{{\rm{cat}}}+\left|{e}_{{\rm{ani}}}-{e}_{{\rm{cat}}}\right|\right)\right)2{\rm{ \% }}$
  or,
- criterion Ib: when the sum of all anions is greater than the sum of all cations: *e*_ani_ > *e*_cat_

However, when criterion Ib is met and the charge imbalance exceeds the estimated analytical uncertainty, the results should be carefully interpreted, as described by Steiger and Heritage^32^. The evaluation is dependent on the specific analytical method, and one should determine if the contribution of the anions in excess is important compared to the total charge imbalance, while considering the analytical uncertainty, this evaluation is carried out in the technical validation section.

When either of the two criteria comply, an equal adjustment to all ions is applied to reach charge balance, by:

4 $${e}_{i,{\rm{a}}{\rm{d}}{\rm{j}}}=\frac{{e}_{i}({e}_{{\rm{c}}{\rm{a}}{\rm{t}}}+{e}_{{\rm{a}}{\rm{n}}{\rm{i}}})}{2\{\begin{array}{c}i\,{\rm{i}}{\rm{s}}\,{\rm{a}}\,{\rm{c}}{\rm{a}}{\rm{t}}{\rm{i}}{\rm{o}}{\rm{n}}\,\,{e}_{{\rm{c}}{\rm{a}}{\rm{t}}}\\ i\,{\rm{i}}{\rm{s}}\,{\rm{a}}{\rm{n}}\,{\rm{a}}{\rm{n}}{\rm{i}}{\rm{o}}{\rm{n}}\,\,{e}_{{\rm{a}}{\rm{n}}{\rm{i}}}\end{array}\}}$$

where *e*_*i*,adj_ is the adjusted and balanced concentration of the single ion, *e*_*i*_ is the initial charge equivalent content of either the single cation or anion to be corrected, *e*_cat_ and *e*_ani_ are the sum of respectively all cations and all anions. In the denominator the sum of either cations or anions is considered in the equation for the adjustment of respectively the individual cation or anion in question (*e*_*i*_), all as Eq/kg.

If both criteria Ia and Ib are not met, pathway II is followed.

#### Pathway II

Pathway II is considered when the following criterion is met:

- criterion II: the excess of cations is greater than 2% compared to the sum of all cations: ∆*e* > *e*_cat_2%

When the charge imbalance is associated with an undetected anion, it is assumed that the excess is related to the least soluble theoretical solids in sequence of their solubility. The balancing procedure abstains from considering the rare contribution to the total charge balance by fluoride, phosphate, oxalate, ammonia, acetate, or formate as partly described by Arnold and Zehnder^33^. Here forth a reasonable assumption can be made to adjust the calcium and magnesium content. Calcium carbonate or calcium hydroxide is present in nearly every lime-based building material, and it is partly dissolved by the extraction procedure. In certain cases, this is also applicable for magnesium carbonate. However, in the intact material these solids cannot go in solution as there is much less water available in the pores to dissolve them compared to what is available in the extraction procedure. This means that there is a maximum excess that can be adjusted reasonably, which is specific to the analytical procedure.

The above reasoning can thus follow the rule of the solubility of possible solids within the extracted solution; specifically, the lowest soluble salt in the mixture will crystallize first, which are^34^: calcite (CaCO_3_): 0.0005 mol/kg, portlandite (Ca(OH)_2_): 0.02 mol/kg, followed by nesquehonite (MgCO_3_.3H_2_O): 0.01 mol/kg. Thus, the adjustment of calcium is carried out until equilibrium is reached, limited to zero calcium content, followed by an adjustment of magnesium. More specifically, when charge balance in excess surpasses the calcium content (*e*_Ca_) the adjustment continues to magnesium (*e*_Mg_). Any additional excess is related to carbonate salts that have a much higher solubility and are relevant within the mixture behavior. Consequently, the following cation associated with carbonates in the sequence of solubility is sodium, and then potassium. These salts are two to three orders of magnitude more soluble compared to calcium and magnesium carbonates; thus, the solubility values of these single salts are not shown as the solubility changes are more relevant within the mixture compositions. The equations below are carried in sequence until charge balance is reached:

5 $$\begin{array}{lll}{e}_{i}^{* } & = & \left\{\begin{array}{c}{e}_{i}-\Delta e\ge 0={e}_{i}-\Delta e\\ {e}_{i}-\Delta e < 0=0\end{array}\right\}\\ \Delta {e}_{i} & = & \left\{\begin{array}{c}{e}_{i}-\Delta e\ge 0=0\\ {e}_{i}-\Delta e < 0=\left|{e}_{i}-\Delta e\right|\end{array}\right\}\end{array}$$

$$\begin{array}{llll}{e}_{i,{\rm{adj}}} & = & \left\{\begin{array}{c}{e}_{{\rm{Ca}},{\rm{adj}}}={e}_{{\rm{Ca}}}^{* }\\ {e}_{{\rm{Mg}},{\rm{adj}}}={e}_{{\rm{Mg}}}^{* }-\Delta {e}_{{\rm{Ca}}}\\ {e}_{{\rm{Na}},{\rm{adj}}}={e}_{{\rm{Na}}}^{* }-\Delta {e}_{{\rm{Mg}}}\\ {e}_{{\rm{K}},{\rm{adj}}}={e}_{{\rm{K}}}^{* }-\Delta {e}_{{\rm{Na}}}\end{array}\right\} & \begin{array}{l}\left(\,\,(5{\rm{a}}\right)\\ \left(\,\,(5{\rm{b}}\right)\\ \left(\,\,(5{\rm{c}}\right)\\ \left(\,\,(5{\rm{d}}\right)\end{array}\end{array}$$

Here *e*_i_^*^ is the adjusted ion content (limited to 0) considering the charge balance in excess (Δ*e*), applicable to the initial ion content (*e*_*i*_) in (Eq. 5). When the excess exceeds the ion content in question the adjustment continues with the difference (Δ*e*_*i*_), following the sequence calcium, magnesium, sodium and potassium until anions and cations are balanced, thus *e*_*i*,adj_ is the final adjusted ion content, all as Eq/kg.

### Determination of gypsum and balanced outputs

With the results (*e*_*i*,adj_) the theoretical content of gypsum is determined for appropriate interpretation of the results, and this, because gypsum is often present in high quantities and overshadows the importance of more soluble salts. This procedure also allows the removed equimolar contents of calcium and sulfate from the data, when required for modeling. For example, the ECOS/Runsalt model does not compute the full range of calculations when including equimolar contents of calcium and sulfate, in the mixture. In fact, ECOS can only handle solutions with six ions, including either Ca^2+^ or SO_4_^2−^ in the system with Na^+^, K^+^, Mg^2+^, Cl^−^, NO_3_^−^.

The above reasoning is grounded on the basis that gypsum overshadows other important solids in the mixture and its low solubility. Seen that gypsum (CaSO_4_.2H_2_O) has a low solubility (0.015 mol/kg^34^) a reasonable assumption can be made that gypsum will crystallize rapidly from any system during evaporation, the latter as described by Clegg and Brimblecombe^30^. Although gypsum is presumed less relevant to influence the mixture behavior under environmental changes^35^, some questions remain unanswered as described by Charola *et al*.^36^. Thus, it remains crucial to determine and evaluate the gypsum content as the salt remains highly important as a primary cause of deterioration, especially in the presence of liquid water.

The determination and exclusion of gypsum is thus necessary for 1) general interpretation of the results and 2) for ECOS to compute, noting that this step has no bearing on the balance between anions and cations. The following equation is considered to determine and evaluate the theoretical gypsum content in the sample:

6 $${e}_{{{\rm{lim}}}_{{{\rm{CaSO}}}_{4}}}=0.5\left({e}_{{\rm{Ca}},{\rm{adj}}}+{e}_{{{\rm{SO}}}_{{4}^{,{\rm{adj}}}}}-\left|{e}_{{\rm{Ca}},{\rm{adj}}}-{e}_{{{\rm{SO}}}_{{4}^{,{\rm{adj}}}}}\right|\right)$$

where *e*_lim,CaSO4_ is the limited content of Ca and SO_4_ in Eq/kg to determine the theoretical gypsum content; *e*_ca,adj_ and *e*_SO4,adj_ are the (adjusted) calcium and sulfate content from Eqs. 4 and 5.

The removal of the theoretical gypsum content is carried out on the individual calcium and sulfate ions, by:

7 $$\begin{array}{c}{e}_{{\rm{Ca}},{\rm{adj}},{\rm{f}}}={e}_{{\rm{Ca}},{\rm{adj}}}-{e}_{{{\rm{lim}}}_{{{\rm{CaSO}}}_{4}}}\\ {e}_{{{\rm{SO}}}_{4},{\rm{adj}},{\rm{f}}}={e}_{{{\rm{SO}}}_{4},{\rm{adj}}}-{e}_{{{\rm{lim}}}_{{{\rm{CaSO}}}_{4}}}\end{array}$$

here, *e*_Ca,adj,f_ and *e*_SO4,adj,f_ are the final adjusted calcium and sulfate contents.

After the removal of gypsum either calcium or sulfate are always zero.

To allow the mutual comparison between different mixture compositions derived from samples with a different weight, the data is normalized, under the condition that the excess (Δ*e*) (as a control) is less than or equal to 1 × 10^−12^ Eq/kg. This value was selected to eliminate rounding errors without implications to data interpretation. The anion and cation values after removal of the theoretical gypsum content as mole fraction (*x*_*i*,adj_) is determined by:

8 $${x}_{i,{\rm{adj}}}=\frac{\frac{{e}_{i,{\rm{adj}}}}{\left|{z}_{i}\right|}}{{\sum }_{k=1}^{n}\frac{{e}_{k,{\rm{adj}}}}{\left|{z}_{k}\right|}}$$

The ion concentrations as mole fraction can be used as direct input for, amongst others, the ECOS/Runsalt model without the need for ambiguous corrections. Furthermore, batch model analysis from different samples can be used for statistical determinations of common ions and salt mixtures found in the built environment, following an approach that was suggested in 2014^37^. Several considerations before using the processed data remain important and are further described.

## Data Records

The data records are available through Z*enodo*^38^. The contents of version 2 include the full integrated database (11412 samples) and charge balance calculation sheet, including raw ion concentrations and balanced outputs (.xlsx), in which each row represents an individual sample. Additionally, an example database (25 mixtures) is included (.xlsx). The database includes metadata, calculation abbreviations and a flowchart.

## Technical Validation

**Fig. 2:**
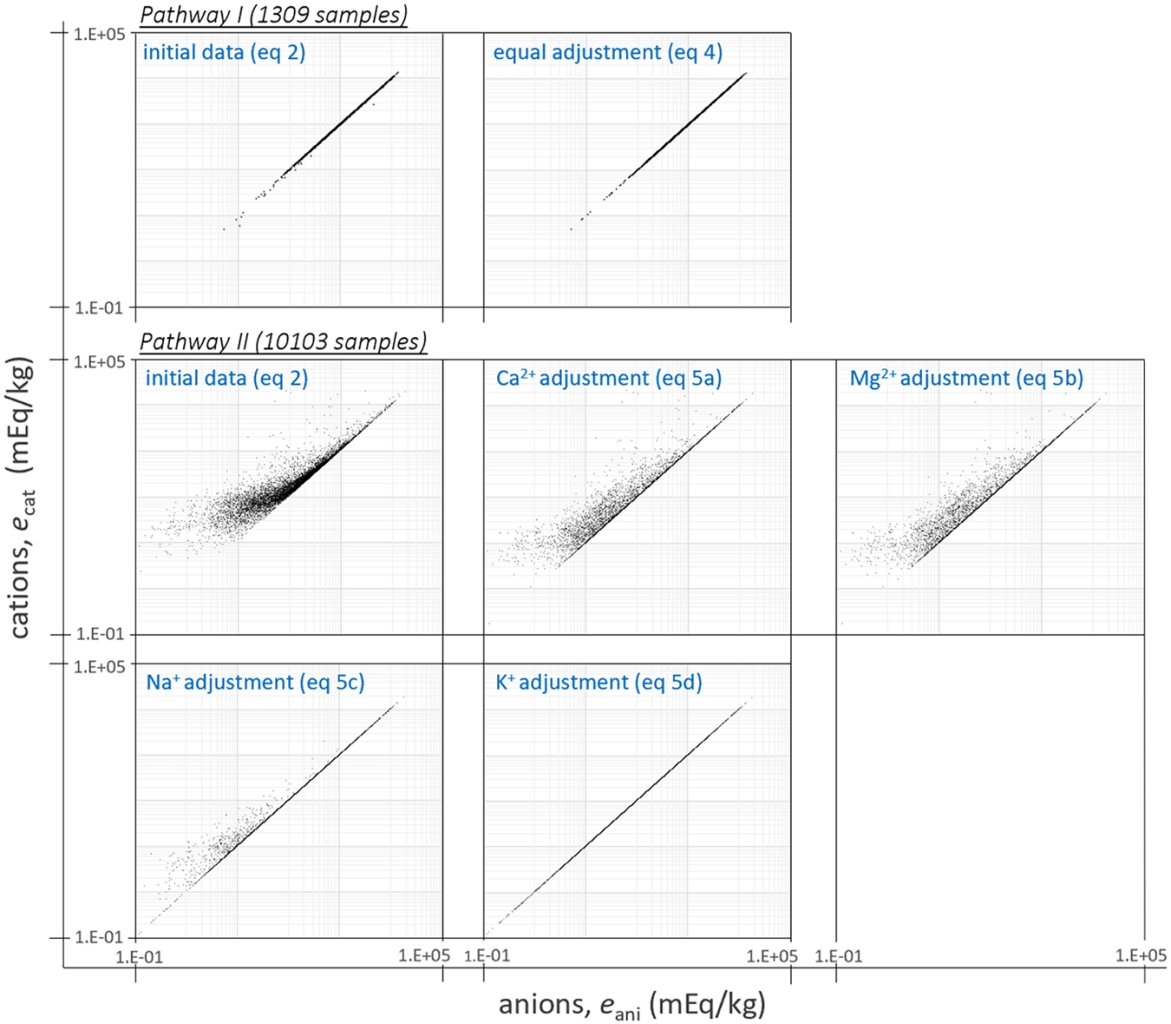
Charge balance sequence in logscale with all anions on the x-axis and all cations on the y-axis (mEq/kg). Top left: initial data (Eq. 2) of samples following pathway I (1309 samples), top right: equal adjustment of all ions (Eq. 4). Middle left: initial data (Eq. 2) of samples following pathway II (10103 samples): middle center: after calcium adjustment (Eq. 5a), middle right: after magnesium adjustment (Eq. 5b), bottom left: after sodium adjustment (Eq. 5c), and bottom right: after potassium adjustment (Eq. 5d).

The charge balance of all 11412 samples is illustrated in Fig. 2. As a result of the charge balance calculations applied to the large dataset several important considerations can be derived for the further consideration of ion mixtures. The processed data is evaluated and shows that after the applied adjustments three samples contain zero ions, leaving the dataset with 11409 samples.

**Fig. 3:**
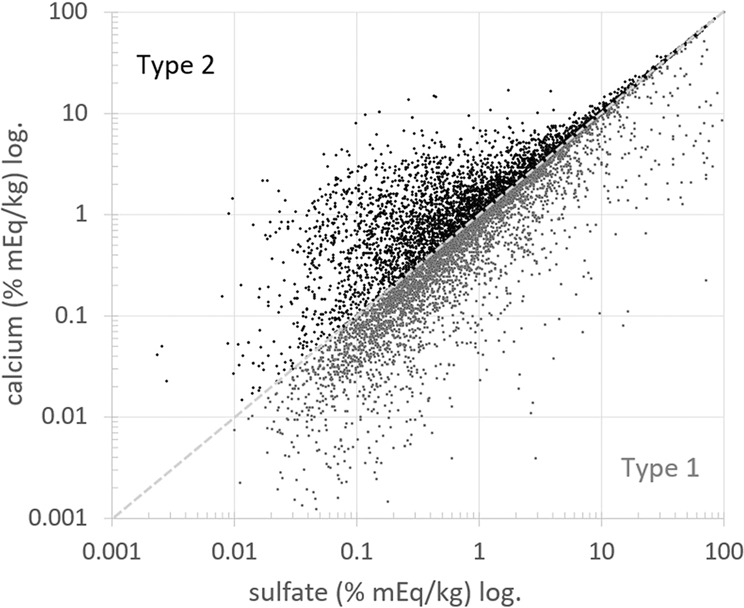
Representation of the ion balance between calcium and sulfate after Eqs. 4 and 5 (% mEq/kg in logscale, as a percentage of the maximum ion value within the dataset per mixture type). Samples including sulfate ions on the x-axis (type 1: 7946 samples) and samples including calcium ions on the y-axis (type 2: 3463 samples), all calcium and sulfate ions that fall together on the dashed diagonal line are equimolar contents of calcium and sulfate, thus the theoretical gypsum content, as determined and removed in Eqs. 6 and 7.

After charge balance is achieved the gypsum content is determined and removed. The removal of equimolar contents of calcium and sulfate follows the assumption that gypsum is mostly present in crystalline form, which is required for the input date of ECOS/Runsalt. Therefore, it is reasonable to assess the balance between both ions (after Eqs. 4 and 5), as illustrated in Fig. 3. All sulfate and calcium ions not balanced, respectively right and left in the figure, are not related to gypsum and belong to different mixture types. Each salt mixture includes either remaining calcium or sulfate ions. This results in two types of mixture compositions typically found in the built environment, as described in^8,39^, and validates the charge balance procedure applied to the dataset. Details on the mixture types, the distribution and their behavior are to be explored further.

**Type 1**) Mixtures including an excess of SO_4_^2−^ ions, with respect to gypsum removal (70% of samples)

**Type 2**) Mixtures including an excess of Ca^2+^ ions, with respect to gypsum removal (30% of samples)

**Fig. 4:**
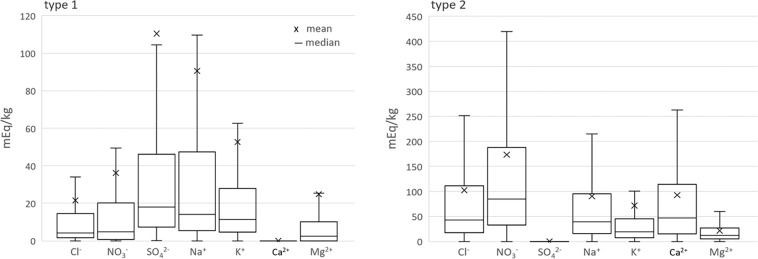
Representation of the most important ions per mixture type derived from the dataset. left: type 1 mixtures (7946 samples), right: type 2 mixtures (3463 samples). Ion content of all samples (mEq/kg) derived from the results after Eq. 7 (excluding equimolar contents of Ca^2+^ and SO_4_^2−^). Boxplots: bottom 25% top 75%, not showing outliers (x 1.5 quartile range).

With the adjusted results in Eq/kg and after the removal of the theoretical gypsum content (Eq. 7) an overview is given of the individual ion content in all samples per mixture type (Fig. 4). In mixtures that are part of type 1 the systems are primarily dominated by sodium and sulfate, followed by potassium and in lesser contents nitrate and chloride ions. The ion ratio in this mixture type 1 is typically less hygroscopic. While in mixtures part of type 2 tend to be more hygroscopic, and are dominated by nitrate, sodium, and chloride, followed by calcium and smaller amounts of potassium ions. Magnesium is in both mixture types the least common ion. The ions that dominate the mixture will significantly influence the mutual crystallization and deliquescence relative humidity, thus the analysis validates the rank of the most important ions that occur in the built environment^8^.

Another important aspect derived from the results is the overall ion content in the two mixture types. With the total salt content (excluding gypsum) translated to weight percent compared to the dry sample mass, the content is 0.9 and 1.1 wt.% for mixture type 1 and 2, respectively. Seen that the majority of samples are taken in areas where salt damage or chromatic alteration^40^ (specifically, moisture stains) are visible a generalized assumption can be made: a total salt content of 1 wt.% (±0.1), excluding gypsum, can be considered a limit value in common building materials, to be further defined. Nevertheless, to avoid misinterpretation after the applied adjustments, and to complete the validation, as par^8,39^, the applied adjustments need to be evaluated further to identify a third mixture composition of interest:

**Type 3)** mixtures containing carbonates (relative within 3% of samples in type 1)

Naturally carbonate rich mixtures within type 2 (including calcium) are excluded after the applied adjustments under Eq. 5a. More specifically, because of the assumption that calcium carbonates will rapidly crystallize from the system due to low solubility. Furthermore, the amount of dissolved calcium carbonates in the solution is depended on the time, and ratio between the amount of pure water added to the dry sample mass to dissolve salts, thus the result will be subject to the individual methodological approach. To avoid error on this front and for completeness of the evaluation, the adjustment of calcium (Eq. 5a) was additionally checked for each sample by evaluating the solubility limit of calcium hydroxide within the specific analytical procedure. Here the solubility of calcium hydroxide instead of calcium carbonate is considered as it is likely that uncarbonated lime is available in the depth of historical lime mortars or recent cement. Accordingly, the theoretical assumption where calcium hydroxide surpassed the solubility limit, was only seen in 0.04% of the samples. In view of the sample size this can be considered a measurement error or the presence of other undetected anions, no further evaluation was carried out at this time.

In the case zero is reached after the adjustment of potassium (Eq. 5d) the sample can be disregarded as no ions are left, excluding perhaps gypsum. This situation occurred for three samples in the entire dataset. Furthermore, the excess contents of calcium, magnesium, sodium, and potassium (Δ*e*_Ca_, Δ*e*_Mg_, Δ*e*_Na_ and Δ*e*_K_, respectively) can be used to calculate a theoretical carbonate content. To allow a proper interpretation of the mixture composition (presented above as mixture types 1, 2 or 3) and to avoid misinterpretation of the total system composition, the degree of each adjustment is calculated to a fraction of the initial cation sum by:

9 $${f}_{\Delta e}=\frac{{e}_{i}-{e}_{i,{\rm{adj}}}}{{e}_{{\rm{cat}}}}$$

where *f*_Δ*e*_ is the amount of substance in excess as a fraction (for calcium, magnesium, sodium, and potassium) compared to the initial sum of cations (before adjustments), *e*_*i*_ is the initial single cation content, *e*_*i*,adj_ is the adjusted single cation content derived from Eq. 5 and *e*_cat_ the initial sum of cations (Eq. 2).

From the result of Eq. 9 and the number of samples for which the ions are equally adjusted (Eq. 4), an overview of the applied corrections can be given (Table 1). With 11.5% of all samples corrected equally (pathway I), while for pathway II calcium was corrected in the majority of samples (88.4%), followed by sodium (17.9%) and less common a correction for magnesium 11.8% and potassium 4.8%. All four cations required correction in 2.1% of the samples.

**Table 1:** Overview of applied adjustments, number and percentage of samples adjusted (total number of samples 11409) following Pathway I (equal adjustment of all ions, Eq. 4) and Pathway II (adjustment of calcium, magnesium, sodium, and/or potassium, Eq. 5).

Sample size 11409	Pathway I	Pathway II			
	Eq. 4 all ions	Eq. 5a Ca^2+^	Eq. 5b Mg^2+^	Eq. 5c Na^+^	Eq. 5d K^+^
Adjustments applied per sample	1309 (11.5%)	10085 (88.4%)	1351 (11.8%)	2044 (17.9%)	552 (4.8%)
	/	1336 (11.7%)		/	
	/	917 (8.0%)			/
	/	234 (2.1%)			

Table 1 presents how often corrections are applicable and specifies the presence of undetected anions in the system. However, the importance of these adjustments in view of the total salt content compared to the sample mass is yet to be determined. The quantity of ions corrected in the mass of the individual sample is equally important to evaluate the probable effect on the mixture composition/behavior. To rank this probable effect, the adjusted ion values are calculated from Eq/kg back to weight fraction, the latter is then compared to the initial ion content.

10 $${w}_{i,{\rm{f}}}=\frac{{e}_{i,{\rm{adj}}}\cdot M}{\left|{z}_{i}\right|}$$

where *w*_*i*,f_ is the final corrected amount of substance as weight fraction per individual ion in the dry sample mass, *e*_*i*,adj_ is the (adjusted) concentration of the ion (Eq/kg), *M* the molar mass (kg/mol) *z*_*i*_ its absolute charge.

**Fig. 5:**
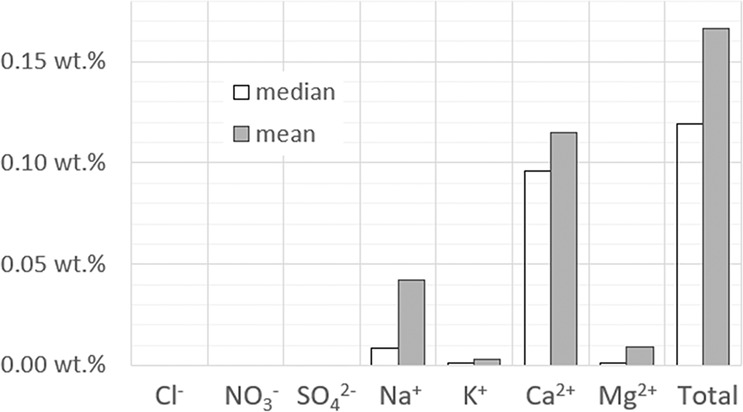
Representation of the adjusted content per ion and the total in wt.% compared to the dry sample mass. Median and mean values of the difference between the initial ion content and the corrected ion content of all 11412 samples are shown.

The results give a more representative indication of the ions corrected in view of the salt content compared to the sample mass. Figure 5 shows the adjusted content of each ion and the total amount after the charge balance calculations.

The adjustments carried out following pathway I are insignificant, which is clearly illustrated by the indistinguishable anion content adjusted at this scale, that is, for the given dataset. After pathway II the adjustments are more important, with calcium being the most adjusted ion at almost 0.1 wt.% (median), followed by sodium at 0.01 (median) and less significant amounts of magnesium and potassium are adjusted. The valuation specifies the limited changes to the systems, yet they are crucial for individual evaluation of sample composition, statistics, and modeling purposes. The individual sample evaluations are described further.

As mentioned earlier the gypsum content plays an important role in the deterioration of building materials yet is considered to have limited influence on the mixture behavior and is thus removed for modeling purposes. However, it remains crucial when giving advice to the field, therefore the theoretical gypsum content is presented separately as weight fraction (*w*_CaSO4_) derived from Eq. 10. In the dataset (11409 samples) the median gypsum content is 0.3 wt.%, while the mean gypsum content is 1.8 wt.%, and excluding 0 the content is 2.6 wt.% (with a standard deviation of 6.3), the latter considering 8100 samples. In view of the determined gypsum content, it is clearly an important salt in building materials which in turn can overshadow more soluble salts.

After the determination of gypsum, the total ion content adjusted (*w*_*tot*,adj_) as a fraction compared to the dry sample mass, excluding gypsum, gives us an overview of the total amount of adjustments applied. The extent of these corrections should be carefully interpreted when compared to visual deterioration patterns *in-situ*. The following equation returns the content of adjustments applied to determine their overall significance compared to the initial ion content:

11 $${w}_{tot,{\rm{adj}}}=\mathop{\sum }\limits_{k=1}^{n}\left({w}_{i}-{w}_{i,{\rm{f}}}\right)-{w}_{{{\rm{CaSO}}}_{4}}$$

The results correspondingly give an indication of how important the theoretical carbonate content is in each sample might be. This is further detailed by calculating the content of corrected Ca^2+^ ions and separately the sum of Na^+^ and K^+^ ions. This allows a better understanding of the less relevant calcium ions likely associated with carbonates and the more relevant magnesium, sodium or potassium ions associated with carbonates in the system. However, it is currently not possible to give a limit value for this assessment. Especially due to the absence of experimental data involving different salt mixture compositions including carbonates to assess the crystallization behavior and damage potential to porous materials. Therefore, an assumption is made based on field experience, i.e., in cases where an excess of cations (excluding calcium) is detected from 0.6 wt.% upwards, carbonate salts such as trona, are regularly detected by XRD-analysis in the efflorescence. In view of this limit value, and possible solids related to carbonates relevant in the pore solution, the calculated number of samples that include ≥0.6 wt.% adjusted sodium, and potassium (sum) is only 1.2% of all samples or 1.8% of all samples in type 1. When considering a lower threshold ≥0.3 wt.%, the number of samples containing possible carbonate salts of importance increases to 2.5 and 3.6%, respectively.

This shows the limited importance of soluble carbonate salts in the presented data. However, these salt mixtures (samples) should be treated with caution when applying the charge balance corrections and attempting to model the crystallization behavior. The same applies to samples for which the total anion content exceeds the total cation content as described earlier in the section ‘charge balance calculations under Pathway I’. In the entire dataset we found that the anion content was in surplus for 41 samples, here Eq. 4 was carried out when criterion Ib was met, for 6 of these sample an excessive correction is observed when evaluating the contribution of the anions in excess to the total charge imbalance. Thus, within the entire dataset not so relevant at 0.05% of all samples, yet crucial for individual evaluation of a salt mixture in a single sample. The full integrated database and charge balance calculation sheet, including raw ion concentrations and balanced outputs are available at^38^.

## Usage Notes

The described charge balance calculations applied to the ion data from 11412 samples follow two main pathways based on the charge imbalance. When an analytical uncertainty is probable, pathway 1 results in an equally adjustment of all ions, which was applicable to approximately one tenth of all samples. When the analytical uncertainty is passed the calculation follow pathway 2 assuming that the measured imbalance is related to undetected anions. Accordingly, cations in surplus are identified in sequence of the solubility of the related solids associated with carbonates. Here, when applicable the initial adjustments correct calcium and magnesium in excess, which are dissolved by the extraction procedure. The following adjustments are carried out for any access related to sodium and potassium. Each of the above ions until charge balance is reached. When using the data and calculations it remains important to understand that the adjustments of sodium and potassium remain relevant within the system behavior. Therefore, when using the dataset, the validation and evaluation remain relevant in all cases. In the dataset presented calcium is naturally the most corrected cation, while sodium is the next most common ion adjusted, followed by the less common adjustments needed for potassium and magnesium.

For modeling purposes, the output data as mole fraction is represented after removal of the theoretical gypsum, if gypsum is required the calculation needs to be adjusted. However, the current method allows the identification of each sample within common mixture types found in the built environment. Type 1 mixtures included remaining sulfate and zero calcium ions. This type contains higher amounts of sodium and sulfate, which renders the mixtures less hygroscopic, other important ions, are potassium and nitrate, followed by lesser contents of chloride and magnesium. Type 2 mixtures with remaining calcium and zero sulfate ions are more hygroscopic mixtures containing considerable amounts of nitrate followed by sodium, chloride, calcium, and lesser contents potassium and magnesium. A third type can be classified as a sub-type within approximately 3% of type 1 mixtures. This sub-type includes carbonates and is only identified by evaluating the content of corrected cations. The identification of this type is important to avoid misinterpretation of the mixture composition and is most relevant when an access of sodium is found, that is, within the given dataset.

The evaluation method is important here as it allows the final comparison between the initial ion content with the corrected values, thus the user can assess the adjustments made in the mixture composition in relation to the mass of the material under investigation. The results of this evaluation show quantitatively the corrections applied to the individual ions, identifying when primarily sodium and potassium are adjusted and caution is necessary. The data as mole fraction can be used as direct input for modeling the crystallization behavior under changing climatic conditions without the need for ambiguous corrections. Thus, permitting batch calculations and statistical analysis of model outputs, enabling future research in the understanding of phase changes in mixed salt systems, evaluation, and prevention of salt deterioration in practice.

## Data Availability

Code is available with the data records through Z*enodo*^38^ (version 2). The contents provide access to the calculations integrated into R, including in- and output of the dataset with descriptors. Specifically, R scripts for charge balance calculations (.R), Full set of raw ion concentrations for the R scripts (.txt), Example of 25 raw ion concentrations for the R scripts (.txt), Full set of balanced outputs from the R script (.txt), Example of 25 balanced outputs from the R script (.txt). The code is available under a CC BY 4.0 license permitting redistribution and reuse with appropriate credit.
